# Anhedonia relates to reduced striatal reward anticipation in depression but not in schizophrenia or bipolar disorder: A transdiagnostic study

**DOI:** 10.3758/s13415-024-01261-1

**Published:** 2025-01-30

**Authors:** Anna Daniels, Sarah A. Wellan, Anne Beck, Susanne Erk, Carolin Wackerhagen, Nina Romanczuk-Seiferth, Kristina Schwarz, Janina I. Schweiger, Andreas Meyer-Lindenberg, Andreas Heinz, Henrik Walter

**Affiliations:** 1 Charité – Universitätsmedizin Berlin, corporate member of Freie Universität Berlin and Humboldt-Universität zu Berlin, Department of Psychiatry and Neurosciences | CCM, Berlin, Germany; 2 Humboldt-Universität zu Berlin, Faculty of Philosophy, Berlin School of Mind and Brain, Berlin, Germany; 3 Health and Medical University Potsdam, Faculty of Health, Potsdam, Germany; 4 MSB Medical School Berlin, Department of Psychology, Berlin, Germany; 5 Technische Universität Dresden, Institute of Clinical Psychology and Psychotherapy, Dresden, Germany; 6 Central Institute of Mental Health, Medical Faculty Mannheim/Heidelberg University, Department of Psychiatry and Psychotherapy, Mannheim, Germany; 7 German Center for Mental Health (DZPG), Partner Site Berlin-Potsdam, Berlin, Germany

**Keywords:** Motivational anhedonia, Reward processing, FMRI, Putamen, Dorsal striatum, Monetary incentive delay paradigm

## Abstract

Anhedonia, i.e., the loss of pleasure or lack of reactivity to reward, is a core symptom of major psychiatric conditions. Altered reward processing in the striatum has been observed across mood and psychotic disorders, but whether anhedonia transdiagnostically contributes to these deficits remains unclear. We investigated associations between self-reported anhedonia and neural activation during reward anticipation and consumption across patients with schizophrenia (SZ), bipolar disorder (BD), major depressive disorder (MD), and healthy controls (HC). Using the Monetary Incentive Delay paradigm, we acquired functional magnetic resonance imaging data sets in 227 participants (18–65 years), including patients with SZ (*n* = 44), BD (*n* = 47), MD (*n* = 56), and HC (*n* = 80). To capture anhedonia, three items of the Symptom Checklist-90-R were entered into exploratory factor analysis, which resulted in a single anhedonia factor. Associations between anhedonia and neural activation were assessed within a striatal region-of-interest and exploratorily across the whole brain (*p*_FWE_ < .05). Self-reported anhedonia was high in MD, low in HC, and intermediate in SZ and BD. During reward anticipation, anhedonia correlated with reduced striatal activation; however, the correlation depended on diagnostic group. Specifically, the effect was driven by a negative relationship between anhedonia and dorsal striatal (putamen) activity within the MD group; for reward consumption, no correlations were found. Our results indicate that anticipatory anhedonia in MD may relate to reduced behavioral motivation via disrupted encoding of motor plans in the dorsal striatum. Future transdiagnostic research should stratify participants by anhedonia levels to achieve more homogeneous samples in terms of underlying neurobiology.

**Supplementary Information:**

The online version contains supplementary material available at 10.3758/s13415-024-01261-1.

Anhedonia, i.e., the loss of pleasure or lack of reactivity to reward (Pizzagalli, 2022), is a core symptom of major psychiatric conditions. The presence of anhedonia predicts worse treatment outcome in major depressive disorder (MD) (McMakin et al., 2012; Uher et al., 2012), poor functional outcome in schizophrenia (SZ) (Kiwanuka et al., 2014), and is transdiagnostically linked to increased risk of suicidality independently of depression (Ducasse et al., 2018). This poor prognosis stands in strong contrast to the limited pharmacological (Klein et al., 2022) and psychological (Sandman & Craske, 2022) treatment options available to specifically alleviate symptoms of anhedonia. Recent conceptualizations of anhedonia stress the importance of distinguishing between different subfacets of anhedonia (Rømer Thomsen et al., 2015; Treadway & Zald, 2011). In this view, anhedonic symptoms can result from disruptions at various stages of the reward process, including deficits in wanting (motivational process of incentive salience), choosing (cost/benefit decision-making), liking (core reactions to hedonic impact), and/or learning (Pavlovian or instrumental associations and cognitive representations) (Rømer Thomsen et al., 2015; Treadway & Zald, 2011). These components involve partially dissociable neurobiological correlates (Berridge & Robinson, 2003; Treadway & Zald, 2011). Developing effective treatments for anhedonia requires a better understanding of these different subfacets and their neurobiological correlates (Kieslich et al., 2022).

Conceptualizing anhedonia as specific deficits in reward processing builds upon existing knowledge of the reward circuitry in animals and humans. From the ventral tegmental area/substantia nigra, the dopaminergic mesocorticolimbic pathway projects to the ventral (VS) and dorsal striatum (DS), and then flows to different parts of the medial prefrontal and cingulate cortex, which project back to the basal ganglia (Haber & Knutson, 2010). Within this circuitry, the VS plays a major role, broadly implicated in the initial encoding of reward properties, and then propagating this information to the DS, which translates reward value into action (Balleine et al., 2007; Haber & Knutson, 2010). In humans, dysfunctions of the reward circuitry, particularly the striatum, have been extensively studied in mood and psychotic disorders. In both MD and SZ, wanting or reward anticipation may be more severely affected, while liking or reward consumption may remain intact (Admon & Pizzagalli, 2015; Kring & Barch, 2014; Lambert et al., 2018; Schmidt et al., 2001; Whitton et al., 2015). In bipolar disorder (BD), the role of altered reward processing is less clear, with hypo- as well as hyperresponsivity to reward-relevant cues observed dependent on mood state and subtype (Nusslock & Alloy, 2017; Whitton et al., 2015). To investigate whether common or distinct processes contribute to these reward deficits across psychiatric conditions, transdiagnostic studies are needed. Specific symptom dimensions, such as anhedonia, are more likely linked to specific neurobiological markers than diagnostic categories (Der-Avakian & Markou, 2012; Insel et al., 2010) and bear transdiagnostic relevance (Heinz et al., 1994; Husain & Roiser, 2018). Nevertheless, only a handful of transdiagnostic studies specifically address neural correlates of anhedonia (Keren et al., 2018; Rømer Thomsen et al., 2015).

To date, transdiagnostic studies of the relationship between anhedonia and neural activation during reward processing yield mixed findings. One well-established paradigm to measure reward anticipation and consumption is the Monetary Incentive Delay paradigm (MID) (Knutson et al., 2001), which robustly activates the striatum. One of the first transdiagnostic studies using the MID finds an association between reduced VS activity during reward anticipation and heightened depressive symptoms across patients with SZ, MD, alcohol disorder, attention deficit/hyperactivity disorder, and healthy controls (HC) (Hägele et al., 2015). Here, anhedonia was not specifically addressed. Reduced VS activity during reward anticipation of the MID is also linked to affective instability in a diverse sample of patients with SZ, BD, MD, autism spectrum disorder, and HC (Schwarz et al., 2020). Note that this sample partly overlaps with the one investigated in the present study, although a different task was used. However, some studies using variants of the MID in mixed samples of patients with mood and psychotic disorders report a lack of transdiagnostic associations of striatal activation during reward processing and anhedonia/depressive symptoms (Arrondo et al., 2015; Wakatsuki et al., 2022) or related symptomatology such as apathy (Kirschner et al., 2020). Considering other reward processing tasks, reduced VS activity during reward consumption is related to increased depressive and anhedonic symptoms across patients with BD and MD (Satterthwaite et al., 2015), but null correlations in a study with a similar design are also reported (Redlich et al., 2015).

Given these mixed findings on the role of anhedonic symptoms in striatal reward processing across mood and psychotic disorders, the purpose of the present study was to specifically investigate the transdiagnostic relationship between anhedonia and the reward circuit during reward anticipation and, exploratorily, during reward consumption. We reanalyzed existing data of a large transdiagnostic sample of patients with SZ, BD, MD, and HC. Compared with our recent publication (Schwarz et al., 2022), we explicitly focused on neural correlates of self-reported anhedonia and used a new preprocessing pipeline (Waller et al., 2022). We expected that reduced striatal activation during reward anticipation and consumption would relate to increased self-reported anhedonia but that the strength of this relationship would vary according to group membership, supporting a joint dimensional-traditional diagnostic approach.

## Materials and methods

### Participants

A total of 362 participants (aged 18–65 years), including patients with SZ (*n* = 89), BD (*n* = 83), MD (*n* = 85), and HC (*n* = 105) were recruited from 2014–2019 at the Central Institute of Mental Health in Mannheim and the Department of Psychiatry and Neurosciences at Charité—Universitätsmedizin Berlin (for previously published reports, see Schwarz et al., 2020, 2022). The respective ethic committees at each site approved the study protocols, and the study was performed in accordance with the Declaration of Helsinki. All participants provided written informed consent. The Structured Clinical Interview for DSM-IV-TR Axis I disorders (SCID-IV German version) (First et al., 2002) was administered by trained clinical interviewers to verify psychiatric diagnoses. Potential medication effects (Abler et al., 2007; Stoy et al., 2012) were addressed as previously described (Schwarz et al., 2022), and results did not correlate with chlorpromazine dose equivalents (CPZ-e) (Davis & Chen, 2004) or an index of medication load (Hassel et al., 2008) (see *Supplemental Methods*). Clinical assessments included the Symptom Checklist 90-Revised (SCL-90-R) (Derogatis, 1994), the Beck Depression Inventory (BDI) (Beck et al., 1961, 1996), the Hamilton Depression Rating Scale (HAM-D) (Hamilton, 1967), the Positive and Negative Syndrome Scale (PANSS) (Kay et al., 1987), and the Young Mania Rating Scale (YMRS) (Young et al., 1978) (Table 1). Healthy controls had no personal or familial history of lifetime axis I disorders; exclusion criteria for patients were a lifetime diagnosis of substance dependence or personality disorder. None of the participants reported a history of neurological or severe medical conditions, a history of head trauma, or any contraindication for functional magnetic resonance imaging (fMRI) (e.g., pregnancy). After quality control, the final sample consisted of 227 participants (see *Supplemental Methods*). Note that additionally missing questionnaire data led to varying sample sizes for each of the analyses, which are respectively specified below.

**Table 1:** Sociodemographic and clinical characteristics for included participants (*n* = 227)

Variable	HC (*n* = 80)	SZ (*n* = 44)	BD (*n* = 47)	MD (*n* = 56)	*F* / *χ*^*2*^
Age: *M*(*SD*)	31.10 (11.10)	33.34 (9.07)	35.02 (10.87)	35.32 (11.45)	2.16, *p* = .09
Gender: f / m	44 / 36	16 / 28	23 / 24	36 / 20	8.15, *p* < .05
Site: Berlin / Mannheim	46 / 34	26 / 18	26 / 21	29 / 27	0.66, *p* = .88
Education (years): *M*(*SD*)	11.96 (1.23)	11.61 (1.47)	12.31 (1.04)	11.74 (1.48)	0.52, *p* = .67
NA	33	21	11	13	
Handedness: right / left / ambi	74 / 4 / 1	35 / 6 / 2	39 / 2 / 6	44 / 5 / 5	12.08, * p *= .06
NA	0	1	0	2	
Medicated: yes / no	-	38 / 5	45 / 2	40 / 15	11.03, * p* < .01
NA		1	0	1	
Smoking: yes / no	12 / 65	32 / 8	20 / 25	14 / 38	50.50, * p* < .0001
NA	3	4	2	4	
Anhedonia factor: *M*(*SD*)	− 0.68 (0.23)	− 0.07 (0.64)	0.19 (1.20)	0.63 (0.92)	37.65, * p* < .0001
NA	3	2	0	0	
BDI: *M*(*SD*)	1.75 (2.50)	11.55 (7.46)	14.60 (9.83)	21.93 (11.62)	70.92, *p* < .0001
NA	0	0	0	1	
PANSS positive symptoms: *M*(*SD*)	-	12.62 (5.42)	8.31 (2.33)	8.51 (2.86)	19.26, *p* < .0001
NA		2	2	1	
PANSS negative symptoms: *M*(*SD*)	-	14.07 (7.42)	10.51 (4.28)	10.16 (4.17)	7.28, *p* < .001
NA		2	2	1	
YMRS: *M*(*SD*)	-	1.10 (3.05)	3.67 (4.41)	0.62 (1.24)	13.02, * p* < .0001
NA		3	2	3	
HAM-D: *M*(*SD*)	-	6.85 (4.00)	6.45 (6.64)	12.87 (6.90)	16.71, * p* < .0001
NA		5	7	2	
Win RT: *M*(*SD*)	231.10 (62.57)	270.30 (68.01)	254.70 (61.95)	257.60 (81.73)	4.93, *p* < .01
Win hit rate: *M*(*SD*)	0.67 (0.14)	0.64 (0.17)	0.63 (0.18)	0.64 (0.16)	0.91, *p* = .43
Monetary win, €: *M*(*SD*)	8.10 (4.11)	6.05 (4.95)	7.40 (3.87)	6.50 (4.52)	2.70, *p* = .05
Movement during scanning, FD: *M*(*SD*)	0.16 (0.05)	0.21 (0.07)	0.17 (0.05)	0.18 (0.06)	6.41, *p* < .001

Anhedonia factor refers to regression scores (mean = 0) obtained from exploratory factor analysis (see *Supplemental Methods*). Number of missing data points stated where missing data occurred. Ambi = ambidextrous; BD = patients with bipolar disorder; BDI = Beck Depression Inventory; FD = framewise displacement; HC = healthy controls; HAM-D = Hamilton Depression Rating Scale; M = mean; MD = patients with major depressive disorder; NA = not available; PANSS = Positive and Negative Syndrome Scale; RT = reaction time; SD = standard deviation; SZ = patients with schizophrenia; YMRS = Young Mania Rating Scale.

### Anhedonia measure

The present study involved a reanalysis of existing data, which was not initially designed with a primary focus on anhedonia, but rather on a broader examination of psychopathology. As a result, no specific, standalone measures on anhedonia were available. To address this, we created an anhedonia subscale by selecting three items from the established depression subscale of the SCL-90-R (#5: “Loss of sexual interest or pleasure”; #14: “Feeling low in energy or slowed down”; #32: “Feeling no interest in things”), following approaches used in previous studies with the BDI (Pizzagalli et al., 2005; Treadway et al., 2009; Winer et al., 2014) and the SCL-90-R (Yang et al., 2020). These items correspond to the wanting subcomponent of anhedonia, reflecting decreased anticipatory pleasure. We chose items from the SCL-90-R, because it is appropriate for community and psychiatric samples, enabling a dimensional approach (Derogatis & Unger, 2010). To examine whether these items loaded onto a single factor, they were entered into exploratory factor analysis (EFA) in the full sample of 362 participants. Exploratory factor analysis was chosen as a suitable method for creating scores when an underlying latent construct is assumed. While using a larger number of items is preferable, factors with three items can be interpreted (Yong & Pearce, 2013). Before conducting EFA, we tested whether our data was suitable by confirming sufficient inter-item correlation and sampling adequacy to produce distinct and reliable factors (see *Supplemental Methods*). A single factor emerged, explaining 51% of the variance, and the factor loadings were all above .55, well above the recommended value of .4 (Field et al., 2012) (*Supplemental* Table S2). The internal consistency was good, especially considering the short length of the scale, with Cronbach’s alpha of .75. Individual factor scores extracted with the regression method were subjected to further analyses.

### Reward fMRI paradigm, fMRI data acquisition, and preprocessing

The MID paradigm (Knutson et al., 2001) in a well-established adapted version (Kirsch et al., 2003; Plichta et al., 2012) was implemented to probe reward anticipation and consumption during fMRI (see *Supplemental Methods*). Note that this version of the MID was primarily designed and validated for the reward anticipation phase, so the results obtained for the reward consumption phase should be viewed as exploratory and thus interpreted with caution. Owing to the nature of the current study, which was a secondary data analysis, no other dedicated measure for reward consumption during fMRI was available. MRI data were collected using matching 3 T Siemens Trio scanners (Erlangen, Germany) in Berlin and Mannheim using identical protocols (see *Supplemental Methods*). Preprocessing was performed by using HALFpipe version 1.2.1 (Waller et al., 2022) that implements fMRIPrep (Esteban et al., 2019) (see *Supplemental Methods*).

### First-level analyses

At the participant level, two different general linear models (GLM) were constructed for the analysis of reward anticipation and consumption, respectively, using FSL FILM (Woolrich et al., 2001) as implemented in HALFpipe (Waller et al., 2022) (see *Supplemental Methods*). Contrasts of interest were the anticipation of monetary salient events relative to control events (contrast: [win anticipation + loss avoidance anticipation] > [anticipation of verbal feedback (“Fast response” or “Too slow”) + neutral anticipation (no win, loss, or verbal feedback)]) (Esslinger et al., 2012; Grimm et al., 2012; Schwarz et al., 2022), and the experience of successful relative to unsuccessful outcomes (contrast: [win hit + loss avoidance] > [win miss + loss]). The rationale for combining win and loss avoidance trials was to increase reliability and statistical power due to the larger number of trials. Moreover, recent meta-analyses have demonstrated neural similarities between win and loss avoidance anticipation within the striatum (Chen et al., 2022; Oldham et al., 2018), suggesting functional equivalence of these trials in the context of the MID (Chen et al., 2022). Exploratory results for additional contrasts (win anticipation > neutral anticipation; win anticipation > implicit baseline; win hit > implicit baseline) are provided in the *Supplement*.

### Group-level analyses

For each contrast, group-level analyses were performed with mixed-effects models in FSL FLAME (Woolrich et al., 2004) as implemented in HALFpipe (Waller et al., 2022). FLAME considers the within-subject variance of lower-level estimates (Woolrich et al., 2004) and provides conservative tests (Eklund et al., 2016). One-sample *t*-tests were performed to confirm striatal activation during anticipation and consumption, and a factorial design with group as covariate of interest, and age, gender, and site as covariates of no interest was specified to test for group differences. To investigate whether the association between anhedonia and striatal activation during reward processing was influenced by diagnosis, an interaction effect between anhedonia and group membership was specified. Specifically, linear regression models were defined that included group, anhedonia, and their interaction as covariates of interest and the above-named covariates of no interest. To evaluate unconditional effects, the model was reestimated excluding the interaction term (Hayes, 2022). *Z*-statistical images were thresholded with a cluster-forming threshold of *z* > 3.1 (Woo et al., 2014) and a family-wise error (FWE) corrected cluster significance threshold of *p* < .05 within a striatal region-of-interest (ROI) as well as exploratorily across the whole brain using the FSL cluster function (Jenkinson et al., 2012). The striatal ROI mask was created based on a priori interest in striatal activity (bilateral nucleus accumbens, caudate, putamen) using the Harvard–Oxford subcortical structural atlas for the MNI152NLin2009cAsym template with a 50% probability threshold as provided by TemplateFlow (Ciric et al., 2022). For significant ROI findings, we calculated the percentage of volume overlap between the significant ROI clusters and the functional divisions of the striatum (Schaub et al., 2021): associative striatum (precommissural dorsal caudate, postcommissural caudate, and precommissural dorsal putamen); sensorimotor striatum (postcommissural putamen); and limbic striatum (corresponds to the VS). Any significant clusters for the interaction effect within the striatal ROI were followed up in R version 4.1.1 (R Core Team, 2020) by extracting mean activity within the respective cluster from first-level statistical maps using FSLUTILS (fslmeants) (Jenkinson et al., 2012). Interaction effects were probed using simple effects analysis within the PROCESS macro for R (Hayes, 2022). Additionally, we repeated the interaction analysis of group x anhedonia on the extracted cluster values with robust methods to decrease the influence of potential violations of model assumptions on the results (Field & Wilcox, 2017; Hayes & Cai, 2007) (e.g., possible heteroscedasticity due to different ranges of the anhedonia variable in the different groups).

## Results

### Behavioral results

Sociodemographic and clinical characteristics are reported in Table 1. Self-reported anhedonia scores derived from the EFA were highest in MD patients, low in HC, and intermediate in SZ and BD patients. Across patient groups, self-reported anhedonia correlated positively with clinical ratings of depressive (Spearman’s rho, *r*_s_ = .63, *p* < .0001, *n* = 131) and negative symptoms (*r*_s_ = .21, *p* < .05, *n* = 140), but not with those of positive symptoms (*r*_s_ = .08, *p* = .72, *n* = 140) or symptoms of mania (*r*_s_ = − .07, *p* = .72, *n *= 137) (corrected using the modified Bonferroni procedure by Holm (1979)).

Analysis of task reaction time (RT) and hit rate data showed faster RT and higher hit rate in salient monetary versus control trials. Across groups, self-reported anhedonia correlated positively with RT but not with hit rates. Patients (especially SZ) responded slower than HC, but hit rates were comparable across participants, indicating that they understood the task equally well and that reaction time thresholds were accurately adapted to individual response times. For details, see *Supplemental Results*.

### fMRI results

Findings related to the reward consumption phase are reported in the *Supplemental Results*. Because of the limited interpretability of the reward consumption phase in this version of the MID task, the primary focus of this study is on the results from the reward anticipation phase.

### Task activation and categorical group differences

As reported previously (Schwarz et al., 2022), reward anticipation robustly activated the bilateral striatum. Furthermore, as reported previously (Schwarz et al., 2022), group differences were observed in the bilateral striatum, specifically in the bilateral putamen. Post hoc group comparisons of each patient group versus HC revealed the largest clusters of differences for SZ patients versus HC. Exploratory results for the reward consumption phase and all cluster tables are reported in the *Supplement* (Table S4).

### Interaction analysis: Group x anhedonia

**Fig. 1:**
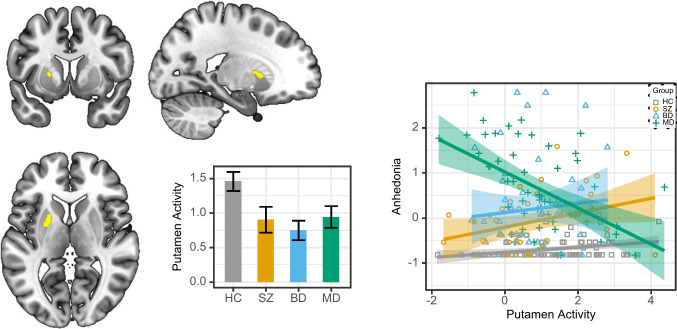
Interaction effect between group and anhedonia in the left putamen. Left panel: cluster of activity in left putamen, displayed at maximum peak of activation (peak [x y z] = [− 20.5 7.5 5.5]; *p*_FWE_ < .05 cluster-corrected within the striatal ROI), and extracted mean activity per group (*z*-values), error bars show standard errors. Right panel: Association between left putamen activity during reward anticipation and anhedonia in each group. BD = patients with bipolar disorder; HC = healthy controls; MD = patients with major depressive disorder; SZ = patients with schizophrenia

For reward anticipation, there was an interaction effect between diagnostic group and anhedonia in the left putamen (68 voxels, *z* = 3.94, *p* = .001, peak [x y z] = [− 20.5 7.5 5.5]; *p*_FWE_ < .05 cluster-corrected within the striatal ROI) (Fig. 1). The ROI cluster had an overlap of 54.41% with the left sensorimotor striatal subdivision, 32.35% with the left associative subdivision, and survived whole brain correction. Further exploratory whole brain results are reported in the Supplement (Table S5). Post hoc simple-effects analysis (controlling for covariates of no interest and corrected for multiple testing using Holm’s approach) showed that the interaction between group and anhedonia was mainly driven by a strong negative relation between left putamen activity and anhedonia in MD patients only (Table 2). There was also a positive relation between left putamen activity and anhedonia in HC, which should be interpreted with caution given that levels of self-reported anhedonia were nonclinical and very low in HC with minimal variance (Table 1). We confirmed that the interaction effect was not solely driven by differences between the MD and HC groups. To test this, we reran the interaction analysis using different reference groups to directly compare the clinical groups (*Supplemental* Table S6). The relationship between anhedonia and left putamen activity was more negative in MD patients compared to each of the other groups, while the effect did not differ between patients with SZ and BD. Exploratorily, for the consumption phase, there were no interaction or main effects within the striatal ROI or across the whole brain at this threshold (see *Supplement*).

**Table 2:** Post-hoc simple effects analysis of the relation between anhedonia and putamen activity within each group (*n* = 222)

Group	Effect	*SE*	*t*	*p*	LLCI	ULCI
HC	1.43	0.54	2.65	< .05	0.37	2.50
SZ	0.60	0.27	2.27	.05	0.08	1.13
BD	0.10	0.16	0.63	.53	−0.21	0.41
MD	−0.63	0.16	−3.92	< .001	−0.95	−0.31

Unstandardized effects and 95% confidence intervals reported. Corrected for multiple testing using Holm’s approach. BD = patients with bipolar disorder; HC = healthy controls; LLCI = lower level of confidence interval; MD = patients with major depressive disorder; SE = standard error; SZ = patients with schizophrenia; ULCI = upper level of confidence interval.

### Sensitivity analyses

Conducting the interaction analysis in an analogous fashion for depression symptom severity as measured by the BDI did not produce any significant clusters within the striatal region of interest during reward anticipation or, exploratorily, during reward consumption.

To evaluate unconditional effects, the model with group and anhedonia as covariates of interest was reestimated excluding the interaction term. A one-sample *t*-test for anhedonia revealed effects in bilateral putamen for reward anticipation (right: 15 voxels, *z* = 4.41, *p* = .044, peak [x y z] = [21.5 3.5 − 2.5]; left: 14 voxels, *z* = 3.82, *p* = .045, peak [x y z] = [− 28.5, 9.5, 3.5]; *p*_FWE_ < .05 cluster-corrected within the striatal ROI). When group was added as a covariate to the model, the effects became nonsignificant.

Additionally, robust methods were applied to the interaction analysis of group x anhedonia on the extracted left putamen cluster values as sensitivity analyses (Field & Wilcox, 2017; Hayes & Cai, 2007). Specifically, standard errors were adjusted using 1) heteroscedasticity-consistent covariance matrix estimators or 2) bootstrapping with 5000 samples, respectively, which yielded results consistent with the output from standard methods. Moreover, given that interaction effects may be confounded by nonlinear effects (Cortina, 1993), we added a quadratic anhedonia term to the model, which also did not significantly alter the results. Finally, the inclusion of medication and smoking status, movement during scanning, and depression symptom severity as measured by the BDI in addition to the above-named covariates of no interest did not significantly change the findings. Among the patient groups, neither CPZ-e nor medication load predicted putamen activity during reward anticipation or anhedonia scores. All model outputs from these sensitivity analyses can be found in the *Supplement* (Tables S7–S11).

## Discussion

Using a large transdiagnostic sample, we assessed the relationship between self-reported anhedonia and striatal activation during reward anticipation and, exploratorily, during reward consumption. We observed a relationship between anhedonia and striatal reward anticipation, which depended on diagnostic group. Specifically, the effect was driven by a negative relationship between anhedonia and left putamen activity during reward anticipation in the group of MD patients; for reward consumption, no correlations were found. The relationship between anhedonia and weaker putamen response to reward anticipation in MD patients was maintained when controlling for relevant confounds, including depression symptom severity and medication status.

During the anticipation of reward, DS reactivity, specifically in the left putamen, was negatively related to self-reported anhedonia in patients with MD. We did not observe significant correlations in the VS during reward anticipation or consumption. One potential explanation of these findings may lie in the functional subdivisions of the striatum, with a gradient between dorsal and ventral areas on the one hand, and anticipatory and consummatory reward processing on the other hand (Schaub et al., 2021; Zhang et al., 2016). While the VS (especially the nucleus accumbens) is thought to primarily code hedonic properties (Haber & Knutson, 2010), the DS (caudate and putamen) plays a role in the learning of specific action-outcome associations and the selection of actions based on their expected reward value (Balleine et al., 2007; Haruno & Kawato, 2006). Of note, the DS is more strongly implicated in tasks where the outcome is contingent upon correct response (O'Doherty et al., 2004), as in the present study, and putamen activity is linked to motivational responding (target RT) during reward anticipation in healthy participants (Staudinger et al., 2011; Takamura et al., 2017). Thus, anhedonia and weaker putamen activity during reward anticipation may be linked by disturbed conversion of motivation into action.

In line with this notion, previous studies connect putamen activity to motor symptoms in MD. Patients with MD with psychomotor disturbances display greater D2 binding potential in the putamen, indicative of less extracellular dopamine (Meyer et al., 2006), as well as reduced functional coupling of the cingulate motor area and the posterior putamen during finger-tapping task performance (Liberg et al., 2014) relative to HC. Another recent study finds a negative link between dopamine transporter availability specifically in the left putamen and psychomotor as well as anhedonic symptoms in patients with MD (D'Onofrio et al., 2024). Psychomotor symptoms and anhedonia are common features of MD (Argyropoulos & Nutt, 2013; Lemke et al., 1999; Stein, 2008) and are thought to occur through disturbances in ascending nigrostriatal and mesolimbic dopaminergic pathways, respectively (Heinz et al., 1994). Possibly in patients with anhedonic MD, the shift from dysfunctions in ventral to more dorsal striatal regions during (anticipatory) reward processing may reflect more of a trait-like marker of the disease. This claim is supported by preliminary evidence that the connection between motivated behavior and neural encoding in the putamen may recover more slowly than value representation in the VS in response to SSRI treatment in MD (Takamura et al., 2017). Taken together with the present findings, anticipatory anhedonia in MD may be hypothesized as reduced behavioral motivation and effort execution to obtain rewards via disruptions in the encoding of motor plans in the putamen.

While the VS is often the focus of studies on anhedonia and reward processing (Husain & Roiser, 2018), several studies provide links between functional and structural putamen alterations and anhedonia or related symptoms. Decreased putamen activity during reward processing is related to depressive symptoms generally (Gotlib et al., 2010; Gradin et al., 2011; Keren et al., 2018; Knutson et al., 2008; Pizzagalli et al., 2009; Robinson et al., 2012; Takamura et al., 2017; Yang et al., 2022; Zhang et al., 2016) and anhedonia specifically (Harvey et al., 2010; Keedwell et al., 2005). Note that putamen hyperreactivity to rewarding stimuli in MD is also reported (Kumari et al., 2003; Mitterschiffthaler et al., 2003; Remijnse et al., 2009), but the overall evidence points towards decreased striatal (including putamen) reward processing in MD, as demonstrated in recent meta-analyses (Keren et al., 2018; Yang et al., 2022; Zhang et al., 2016). Interestingly, several reports emphasize decreased DS volume specifically in MD (Beyer & Krishnan, 2002; Kempton et al., 2011; Koolschijn et al., 2009), and populations at-risk for MD (Pagliaccio et al., 2020; Talati et al., 2022), and decreased putamen volume is related to higher self-reported anhedonia (Auerbach et al., 2017; Sachs-Ericsson et al., 2017; Schaub et al., 2021). To what extent these structural alterations reflect functional deficits requires further investigation, although some studies show relationships between striatal response to reward anticipation and increased putamen volume (Caseras et al., 2013; Yip et al., 2015). Future studies should continue to include DS next to VS ROI to shed further light on their interaction and how disruptions within the striatal system lead to the symptoms of anhedonia.

We did not observe a group-independent dimensional association between anhedonia and striatal activation during reward anticipation or consumption. Previous transdiagnostic studies provide mixed evidence to date of a dimensional link between reduced striatal response to reward and increased depressive and/or anhedonic symptoms. Supporting dimensionality, reduced activation in the VS during reward anticipation is transdiagnostically associated with increased depressive symptoms (Hägele et al., 2015) as well as a factor score reflecting affective instability (Schwarz et al., 2020), but these measures do not reflect anhedonia specifically. Moreover, during unpredictable wins versus losses in a monetary guessing task, weaker activation in the VS robustly correlates with increased depression severity (total BDI) across both unipolar and bipolar depression groups (Satterthwaite et al., 2015). Exploratorily, the anhedonia subscale of the BDI is also related to reduced VS activation across diagnostic groups (Satterthwaite et al., 2015). However, several studies (Arrondo et al., 2015; Kirschner et al., 2020; Redlich et al., 2015; Wakatsuki et al., 2022) using variants of the MID do not observe brain-behavior correlations across diagnostic groups. Depressive and anhedonic symptoms are related to lower VS activation during reward anticipation in patients with SZ but not MD, despite greater self-reported symptom severity in the MD patients compared with the SZ patients (Arrondo et al., 2015). Similarly, a recent study (Kirschner et al., 2020) demonstrates that, although BD and SZ patients have comparable levels of self-reported apathy (which included anhedonia), apathy only relates to weaker striatal activation during reward anticipation in SZ patients. Another recent study does not observe correlations between depression symptom severity and activity in striatal regions during reward anticipation in a mixed sample of BD and MD patients (Wakatsuki et al., 2022). Furthermore, a study using a card-guessing task in patients with BD and MD (Redlich et al., 2015) does not observe any correlation between VS activation and symptoms of depression or anhedonia. In sum, the findings at hand considered together with previous work are in favor of a joint dimensional-traditional diagnostic approach to the role of anhedonia in striatal reward processing.

## Strengths and limitations

In the current study, although anhedonia and depressive symptoms were correlated, the negative relationship between anhedonia and putamen activity during reward anticipation in patients with MD was maintained when controlling for depressive symptoms, and we did not observe any brain-behavior correlations of the BDI within the striatum. This lends support to our interpretation of reduced dorsal striatal activity as a specific marker of anhedonia in MD, which could potentially inform targeted treatment strategies. Conversely, there are limitations to be considered. First, specific, validated anhedonia questionnaires were not available in this data set. Thus, the present results were obtained with factor scores reflecting anhedonia obtained from items measuring current symptoms from the SCL-90-R, potentially inducing a floor effect in the group of HC. Further, while a factor derived from EFA with three items is interpretable, and the factor score derived from the three items most likely reflected anticipatory anhedonia with satisfactory internal consistency, a solution based on a larger number of items would provide greater stability. Recent anhedonia scales, such as the Temporal Experience of Pleasure Scale (Gard et al., 2006), the Dimensional Anhedonia Rating Scale (Rizvi et al., 2015), or the recently developed Positive Valence Systems Scale (Khazanov et al., 2020), provide more comprehensive measures of anhedonia and its different subfacets for dimensional analyses. Future work should include several measures of anhedonia to further disentangle the neural correlates of its anticipatory and consummatory facets. In particular, selecting participants based on more objective behavioral assessments of incentive motivation, such as value-based choice tasks (Berwian et al., 2020; Pizzagalli et al., 2005; Treadway et al., 2009) may yield more homogenous samples in terms of underlying neurobiology, thus allowing direct testing of the proposed link between anhedonia and decreased striatal responding during reward anticipation via disturbed encoding of motivation into action beyond diagnostic categories.

In a similar vein, the lack of correlation in the BD and SZ groups may have been because their striatal deactivation was influenced by disease factors beyond anticipatory anhedonia. Indeed, hedonic deficits can occur through disruptions in any stage of the reward process (Der-Avakian & Markou, 2012; Lambert et al., 2018; Rømer Thomsen et al., 2015), different anhedonic subtypes may be present (Lambert et al., 2018; Rømer Thomsen et al., 2015; Whitton & Pizzagalli, 2022), and similar symptoms do not necessarily indicate similar pathophysiological mechanisms (Whitton et al., 2015). Symptoms, such as cognitive disorganization and aberrant salience processing in SZ (Esslinger et al., 2012; Lambert et al., 2018; Nusslock & Alloy, 2017; Whitton et al., 2015) and (hypo)mania or increased loss sensitivity in BD (Abler et al., 2008; Nusslock & Alloy, 2017; Whitton & Pizzagalli, 2022; Whitton et al., 2015), may contribute to altered reward responsiveness in the striatum. In our sample, levels of positive symptoms were highest in the SZ group, and our BD sample was euthymic, with a low proportion of BD type II patients where depression and reward anticipation deficits may be more prevalent (Whitton et al., 2015). As a related point, although we did control for basic sociodemographic variables in all main analyses and conducted additional sensitivity analyses to account for depressive state, smoking, and medication status, as well as medication load, we cannot rule out that our effects may partly be due to influences of psychotropic medication.

Finally, differences in task design deserve mentioning. The MID version employed used a fixed cue-task interval (no variable interstimulus interval) with one cue per condition; thus, the rewarded task was more predictable than alternative task versions (Becker et al., 2017). While the investigation of both anticipatory and consummatory reward processing is a strength of the current investigation, the task is primarily designed to capture the anticipation phase (Plichta et al., 2012), as target-receipt intervals are not jittered and there are low numbers of trials per outcome type, reducing reliability. Moreover, (ventral) striatal regions respond most strongly with increasing magnitude (Haber & Knutson, 2010; Takamura et al., 2017) and at maximum uncertainty of rewards (50% vs. average success rate of 66% in our study) (Berns et al., 2001), which may have additionally attenuated the present findings for the outcome phase. Nevertheless, because our main findings were obtained for the well-validated anticipation phase, reduced putamen activity when anticipating rewards should be further investigated as a potential marker of anticipatory anhedonia in MD in future research.

## Conclusions

A general anhedonia factor extracted from the SCL-90-R correlated with reduced putamen activity during reward anticipation in patients with MD, but not in patients with SZ, BD, or in HC, possibly reflecting a specific deficit in action-outcome encoding in anhedonic depression. Future studies should continue to investigate different subfacets of anhedonia and consider including samples based on anhedonia score to achieve a broad range of clinical and non-clinical levels of anhedonia across diagnoses. Especially employing laboratory tasks as behavioral indicators of anhedonia during sample selection may yield more homogeneous samples in terms of underlying neurobiology.

## Supplementary Information

Below is the link to the electronic supplementary material.

Supplementary file1 (DOCX 1541 KB)

## Data Availability

Not available.

## Availability of data and materials

None of the data or materials for the experiments reported here is available due to privacy and ethical restrictions.

## Declarations

### Open practices statement

None of the data or materials for the experiments reported here is available due to privacy and ethical restrictions, and none of the experiments was preregistered.
