# Supplementary material for: Anhedonia relates to reduced striatal reward anticipation in depression but not in schizophrenia or bipolar disorder: A transdiagnostic study
Source: Cogn Affect Behav Neurosci. 2025 Jan 30;25(2):501–14. doi: 10.3758/s13415-024-01261-1 (PMC11906564; doi:10.3758/s13415-024-01261-1)
Supplement: Supplementary file 1 — Supplementary file1 (DOCX 1541 KB) [file 13415_2024_1261_MOESM1_ESM.docx]

**Supplementary Information**

Supplementary materials for this study are available at the Open Science Framework (OSF) and can be accessed at the following URL: https://osf.io/m89q7
